# Cost analysis of patients undergoing cardiac surgery managed with or without cerebral oximetry (INVOS)

**DOI:** 10.1186/1753-6561-6-S4-O16

**Published:** 2012-07-09

**Authors:** D Walsh, M Bennett, S Bennett

**Affiliations:** 1 Hull York Medical School, UK; 2 Cardiothoracic Unit, Castle Hill Hospital, UK

## Introduction

INVOS measures cerebral oxygen saturation. A 100 patient audit examining the efficacy of INVOS showed a reduction in mortality and post-operative length of stay (LOS) against national statistics [1]. Our aim was to analyse the cost effectiveness of INVOS in mixed and coronary bypass (CABG) only cardiac surgery by comparing the INVOS group to a control group.

## Methods

A cost analysis of the INVOS group was done by comparing against 100 control patients who underwent surgery immediately prior to the INVOS audit. Hospital finance data was used to calculate costs. The areas for comparison were cost of: INVOS equipment; ICU and non-ICU post-operative LOS; stroke rehabilitation. Previous INVOS studies have focused on CABG only patients [2,3]. In order to compare outcomes with those found in these studies we selected the CABG only patients out of the two groups. This data was then analysed to give a final saving for CABG only patients.

## Results

Despite the equipment cost, all other outcomes had a reduced cost in the INVOS group. Overall there was a saving of £102,000 per 100 patients undergoing cardiac surgery. There was improvement in all outcomes in both INVOS (n=65) and the control group (n=66) once the criteria of CABG only had been applied. LOS was reduced in both groups, and neither group contained an incident of stroke. This resulted in an even greater saving of £114,000 per 100 CABG only patients. Both comparisons, mixed and CABG only, showed the biggest cost saving to be due to a reduction in post-operative LOS both in ICU and on the wards. This correlates with the findings of previous studies [2,3].

## Conclusions

This cost analysis shows a significant saving when using INVOS in the management of both mixed and CABG only cardiac surgery. The CABG only patients showed the greatest saving of £114,000 per 100 patients. Previous literature only considered CABG patients and did not undertake any cost analysis. However, further analysis with mixed cardiac surgery and a randomised trial is required.
